# A Study and Modeling of *Bifidobacterium* and *Bacillus* Coculture Continuous Fermentation under Distal Intestine Simulated Conditions

**DOI:** 10.3390/microorganisms10050929

**Published:** 2022-04-28

**Authors:** Svetlana A. Evdokimova, Boris A. Karetkin, Elena V. Guseva, Maria G. Gordienko, Natalia V. Khabibulina, Victor I. Panfilov, Natalia V. Menshutina, Nina B. Gradova

**Affiliations:** 1 Biotechnology Department, Mendeleev University of Chemical Technology, 125047 Moscow, Russia; evdokimova.s.a@muctr.ru (S.A.E.); khabibulina.n.v@muctr.ru (N.V.K.); vip@muctr.ru (V.I.P.); gradova.n.b@muctr.ru (N.B.G.); 2 Department of Chemical and Pharmaceutical Engineering, Mendeleev University of Chemical Technology, 125047 Moscow, Russia; guseva.e.v@muctr.ru (E.V.G.); gordienko.m.g@muctr.ru (M.G.G.); menshutina.n.v@muctr.ru (N.V.M.)

**Keywords:** *Bifidobacterium adolescentis*, *Bacillus cereus*, oligofructose, probiotics, prebiotics, foodborne pathogens, growth inhibition model, continuous coculture fermentation, computational biology

## Abstract

The diversity and the stability of the microbial community are associated with microecological interactions between its members. Antagonism is one type of interaction, which particularly determines the benefits that probiotics bring to host health by suppressing opportunistic pathogens and microbial contaminants in food. Mathematical models allow for quantitatively predicting intrapopulation relationships. The aim of this study was to create predictive models for bacterial contamination outcomes depending on the probiotic antagonism and prebiotic concentration. This should allow an improvement in the screening of synbiotic composition for preventing gut microbial infections. The functional model (fermentation) was based on a three-stage continuous system, and the distal colon section (N_2_, pH 6.8, flow rate 0.04 h^–1^) was simulated. The strains *Bifidobacterium adolescentis* ATCC 15703 and *Bacillus cereus* ATCC 9634 were chosen as the model probiotic and pathogen. Oligofructose Orafti P95 (OF) was used as the prebiotic at concentrations of 2, 5, 7, 10, 12, and 15 g/L of the medium. In the first stage, the system was inoculated with *Bifidobacterium*, and a dynamic equilibrium (*Bifidobacterium* count, lactic, and acetic acids) was achieved. Then, the system was contaminated with a 3-day *Bacillus* suspension (spores). The microbial count, as well as the concentration of acids and residual carbohydrates, was measured. A *Bacillus* monoculture was studied as a control. The stationary count of *Bacillus* in monoculture was markedly higher. An increase (up to 8 h) in the lag phase was observed for higher prebiotic concentrations. The specific growth rate in the exponential phase varied at different OF concentrations. Thus, the OF concentration influenced two key events of bacterial infection, which together determine when the maximal pathogen count will be reached. The mathematical models were developed, and their accuracies were acceptable for *Bifidobacterium* (relative errors ranging from 1.00% to 2.58%) and *Bacillus* (relative errors ranging from 0.74% to 2.78%) count prediction.

## 1. Introduction

The study of interactions between members of the microbial community is a rather complicated but extremely important task, since most microorganisms naturally enter these systems. The intestinal microbial community has the most significant impact on the health of the host, and the possibility of its modulation by probiotics [1], prebiotics [2], and their synbiotic compositions [3] have been the subject of scientific research over the past few decades. The introduction of new populations into microbiocenosis (in the case of the use of probiotics and synbiotics) can be considered from the point of view of both medicine and microbial ecology. Although the effectiveness of such an intervention, as noted earlier [1,4], can only be finally confirmed in clinical trials, the preliminary investigations in vitro are relevant for a number of reasons.

The main advantage of functional intestine models in vitro is the ability to obtain highly reproducible results due to the exclusion of a number of external factors, as well as the ability to strictly control the parameters. From an ethical point of view, the use of models does not practically require such hard restrictions as trials on humans, and it is much more humane than trials on animals. Moreover, since pharmaceutical procedures and dietary studies usually take a long time, representative models in vitro can significantly speed up the result. Lastly, such studies are less expensive [5,6].

Currently, models are used that simulate not only the conditions of the gastrointestinal tract such as pH, temperature, and atmospheric composition, but also the relationship of the microbiota with cells of the host intestinal epithelium and mucin, as well as the immune reactions. Examples include (1) the Transwell “apical anaerobic model of the intestinal epithelial barrier”, (2) the host–microbiota interaction (HMI) model, (3) the “Human oxygen–Bacteria anaerobic” (HoxBan) system, (4) the human gut on a chip, and (5) the HuMiX model [6]. However, such models are more complex and expensive to implement, since they require the expansion of cell lines such as Caco-2, HT-29, T-84, and DLD-1. On the other hand, three-stage continuous culture systems are widely used to study microbial interactions within the intestinal community.

The three-stage continuous model was firstly proposed by Gibson et al. in 1988 [7]. Later, it was validated as a system of three reactors connected together, which differed in terms of pH and medium replacement frequency. The system corresponded to three sections of the large intestine: proximal, transverse, and distal [8]. Similar systems are widely used to assess the impact of various factors (prebiotic, probiotic, or synbiotic intake, diet, microbial infection, etc.) on the intestinal microbial community. Probert et al. [9] used this model to study and compare the effects of polydextrose, lactitol, and fructooligosaccharides (FOS) on the quantitative and qualitative composition of the microbiota, as well as on the production of various metabolites (short-chain fatty acids, SCFAs). The three-stage continuous system was applied to compare the prebiotic potential of orange juices with the addition of prebiotics [10]. The impact on the microbiota was assessed through an analysis of SCFAs and the microbial profile. A comparison of the enzymatic activity of microbial communities of vegetarians and omnivores (inoculated from the respective donors) and their response to a high-protein diet and prebiotics was also performed on functional models [11]. On a single-stage model simulating the transverse colon, Astó et al. [12] studied the enzymatic activity of intestinal bacteria against fructans with different degrees of polymerization. Members of the microbiota have been shown to be able to metabolize large polysaccharide molecules, breaking them down into monomers available to epithelial cells. The three-stage continuous model was applied for the assessment of *Staphylococcus aureus* contamination of the gut microbiota [13]. Furthermore, in vitro functional intestinal models are used to assess the bioavailability of various substances. Thus, the efficiency of metabolization of plant polyphenols (2-arylbenzofurans) in vitro by members of the microbiota was studied using a batch fermentation model of the human colon, followed by the analysis of the permeability of metabolites into intestinal epithelial cells on the Caco-2 cell line [14].

One modification of the model consisting of a fermenter [15] is intended for studying the proximal section. Controlled parameters are the number of individual groups (genera or phyla) of bacteria, the formation of metabolites (alcohols, short-chain fatty acids, lactic acid), and the composition of the gaseous atmosphere. In the GIS1 single-chamber system of simulation of the human colon, cultivation was carried out in a single vessel with a semi-periodic (fed-batch) medium exchange. The pH in the vessel was changed sequentially, simulating the pH of the ascending (4 h), transverse (8 h), and descending (12 h) sections of the large intestine [16]. Using the GIS1 model, a positive effect was found after the introduction of fungi on the count of *Bifidobacterium* and *Lactobacillus*. It was also shown that human indigestible fungal polyphenols (proanthocyanidins) were metabolized by the microbial community members, resulting in an antioxidant effect [17].

There are various types of interactions in microbial communities: resource competition, metabolic interactions (including cross-feeding and sequential utilization), allelopathy (including the production of microbial chemicals, such as bacteriocins), etc. [18]. It is possible to quantitatively characterize the measure of interaction on the basis of an experiment or a mathematical model. A coculture fermentation of basic members (as a compromise between the reliability of the results and the complexity of the experiments) is commonly applied for data collection. Several types of computational models based on microbial coculture have been introduced to predict the community response to a disturbance and to evaluate manipulation techniques. Ecological models and genome-scale models can be generally distinguished [19]. Usually, the model describes one or, less often, several types of interactions. Microbial competition is described using the coculture models such as the Lotka–Volterra (LV) model and the Baranyi–Roberts model coupled with the Giménez–Dalgaard model (BR–GD) or the Giménez–Dalgaard model coupled with the Huang model (H-GD). Using these models, predictions have been made for the co-growth of pathogens (*Listeria monocytogenes*, *Staphylococcus aureus*, *Escherichia coli*) and lactic acid bacteria (LAB) in artificial media or foods [20,21,22,23,24]. These types of models are most often applied in the area of food safety to predict and prevent microbial spoilage. In particular, the effectiveness of biopreservation by beneficial bacteria can be assessed using the models. The LA model was also applied for deciphering microbial interactions in synthetic human gut microbiome communities [25]. In these models, the measure of influence is the number (concentration) of cells in a given population. The model of sequential utilization of food carbohydrates by human microbiota was developed by Munoz–Tamayo et al. [26]. It takes into account the growth of the biomass of individual metabolic groups, the consumption of substrates, the formation of products (intermediate and final), and mass transfer. Cross-feeding in cocultured human microbial community members for lactate utilization and butyrate production was described using a partial case of the previous model [27]. The kinetic model was applied to predict metabolic interaction in a coculture of four members of the human microbiota [28]. The advantage of this approach is that the quantitative relationships linking microbial growth, substrate consumption, and metabolite production are considered. Moreover, the influence of chemical environmental factors (substrate and metabolite concentrations) on the specific growth rate was described using the Andrews model [29]. It should be noted that data for the development and validation of mathematical models, including those describing the intestinal microbial community, were obtained under static fermentation conditions.

Previously, we proposed and tested, under the conditions of static coculture, a model of the inhibition of pathogen growth by probiotics, in which the measure of interaction (antagonism) is expressed through the production of organic acids (lactic and acetic acids). Such a model allowed us to evaluate the effect of prebiotics [30,31]. However, as noted above, conditions in the intestine correspond to a continuous culture with low dilution rates, which significantly differs from batch culture. The aim of this work was to develop an experimental and mathematical model to describe the inhibition of pathogen growth by probiotics under conditions close to the large intestine. Taking into account the studies described above, two representatives of the microbial community were taken in mono and coculture: *Bifidobacterium* (as a commensal) and *Bacillus cereus* (as a pathogen). The proximal intestine was considered as representative for compiling a mathematical model; accordingly, the cultivation was carried out in one fermenter.

## 2. Materials and Methods

### 2.1. Bacterial Strains and Inoculate Preparation

The strain *Bifidobacterium adolescentis* VKPM AC1662 (corresponding to ATCC 15703) was considered a model probiotic with high β-fructofuranosidase activity [30,32]. The strain *Bacillus cereus* VKPM B8076 (corresponding to ATCC 9634) was used as a model foodborne pathogen. The freeze-dried samples were purchased from the Russian National Collection of Industrial Microorganisms (VKPM, Moscow, Russia) and stored at temperatures not exceeding 8 °C until use.

A carbohydrate-free medium and carbohydrate solution were prepared, sterilized separately, and mixed before inoculation. The carbohydrate-free medium (according to [32] with some modifications) was composed as follows (g/L): meat extract (Panreac, Barcelona, Spain), 5; yeast extract (Springer, Maisons-Alfort, France), 7.6; casein tryptone (Difco Laboratories, Detroit, MI, USA), 10; urea, 2; (NH_4_)_2_SO_4_, 5; MgSO_4_·7H_2_O, 0.2; FeSO_4_·7H_2_O, 0.01; MnSO_4_·7H_2_O, 0.007; NaCl, 0.01; cysteine (all from DiaM, Moscow, Russia), 0.5; Tween-80, 1; ascorbic acid (both from AppliChem, Darmstadt, Germany), 1. The pH was adjusted to 7.0. Oligofructose (OF, Orafti^®^ P95, BENEO-ORAFTI, Tienen, Belgium) was used as a standard prebiotic. A concentrated OF solution in distilled water was prepared. The sterilization was carried out at 115 °C for 30 min.

The strain samples were restored in sterile phosphate-buffered saline (PBS), transferred into tubes with a sterile culture medium, and incubated at 37 °C. The sealed vessels with two branches were supplied with membrane autoclavable vent filters (Midisart 2000 PTFE, 0.2 μm, Sartorius, Goettingen, Germany), and clamps were applied for the inoculate preparation. The vessels were sterilized with a carbohydrate-free medium. The OF solutions were aseptically added to the vessel before inoculation to obtain the same concentration as in the fermentation (see below). Then, the inoculation of the daily culture of *Bifidobacterium* or *Bacillus* was performed in the vessels with immediate sparging by N_2_ (extra pure) through a vent branch which reached the bottom. The inoculates were incubated at 37 °C with shaking (180 rpm) overnight.

### 2.2. Continuous Fermentation

**Figure 1:**
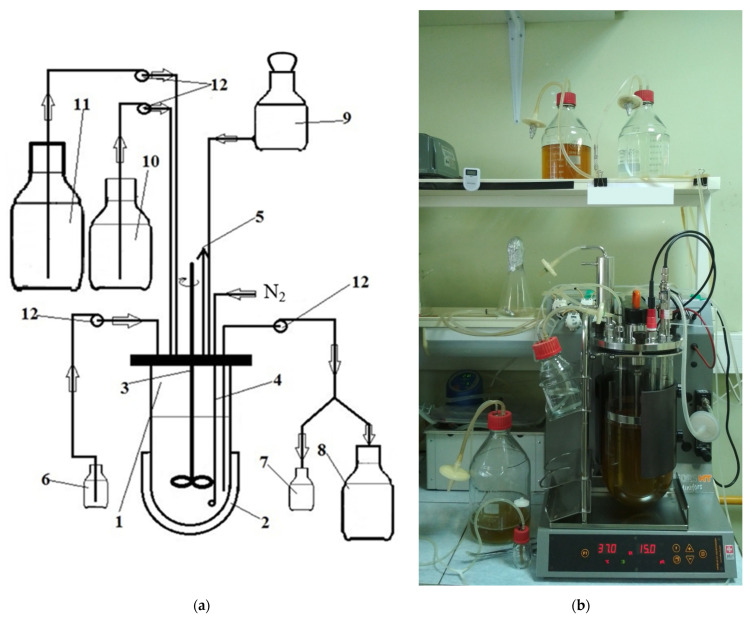
Schematic (**a**) and image (**b**) of the unit used for the simulation of descending colon conditions in vitro. 1—bioreactor, 2—heating jacket, 3—impeller, 4—system of nitrogen supply through a sterilizing filter (0.2 µm), 5—gas exhaust through the sterilizing filter (0.2 µm), 6—titration bottle, 7—sampler, 8—bottle for biosuspension collection, 9—inoculation flask, 10—OF solution bottle, 11—carbohydrate-free medium bottle, 12—peristaltic pumps. All vessels (6, 7, 8, 10, and 11) were supplied with seal cups and sterilizing filters (0.2 µm). The inoculation flask sealed with a plug was clamped after usage.

The fermentations were carried out in a unit (Figure 1) based on a 5 L laboratory bioreactor Minifors (Infors HT, Bottmingen, Switzerland) supplied with pH (405-DPAS-SC-K8S/325, Mettler Toledo, Urdorf, Switzerland) and pO_2_ (Hamilton Company, Reno, NV, USA) sensors. The control block of the bioreactor was provided with four peristaltic pumps, applied for the inflow of carbohydrate-free medium and carbohydrate solution, the outflow of cultural fluid, and the adjustment of pH. The continuous fermentations were carried out at two stages; the monoculture of *Bifidobacterium* was maintained until dynamic equilibrium was reached at the first stage, and the contamination with *Bacillus* spores was performed at the second stage. The bioreactor was sterilized with carbohydrate-free medium. All connections and open-volume manipulations were performed aseptically to avoid contamination. The additives were introduced through a flask in a laminar flow cabinet located in the immediate vicinity of the unit. Since the strains used in the study were not biohazardous (biosafety level 1), the unit was installed on a standard open laboratory bench without the use of special containment equipment. The sterile OF solution was introduced aseptically through the inoculation flask. The concentrations of OF were varied for *Bifidobacterium* monoculture experiments (2, 5, 7, 10, 12, and 15 g/L) and coculture experiments (5, 7, 10, 12, and 15 g/L). The culture medium was sparged with nitrogen (pO_2_ < 0.5). The fermentation conditions corresponding to the descending colon were a temperature of 37 °C, pH of 6.8, and anaerobic atmosphere.

An overnight culture (approximately 16 h) of the *Bifidobacterium* strain was inoculated, and the fermenter was bubbled repeatedly. One hour after inoculation, the sterile carbohydrate-free medium (from bottle 11) and OF solution (from bottle 10) were continuously supplied to the bioreactor in a volume ratio of 2:1, and the cultural fluid was supplied to the bottle for biosuspension collection (8) in an equal volume. The dilution rate was 0.04 h^−1^. The pH was maintained at 6.8 by adding a 20% *w*/*w* solution of sodium hydroxide. The level of dissolved oxygen was controlled by a pO_2_ sensor (<0.5%). To create anaerobic conditions, the fermenter was bubbled with nitrogen (extra pure) at least twice a day. These conditions were considered as close as possible to those of the descending colon [8]. After reaching the dynamic equilibrium, the unit was contaminated with a 3-day (spore) culture of *Bacillus cereus* ATCC 9634. In the control experiment, only the monoculture of *B. cereus* ATCC 9634 was inoculated.

### 2.3. Enumeration of Bacteria

Tenfold serial dilutions in sterile PBS were made and plated on the appropriate medium, and the incubation was carried out at selective conditions for separate enumeration of *Bacillus cereus* and *Bifidobacterium adolescentis*. The dilutions were plated on MRS agar [33] and incubated at 37 °C in the air for *Bacillus* counting. BFM agar [34] was applied for *Bifidobacterium* enumeration. The pH was adjusted to 5.5 by adding propionic acid (5 mL per 1 L of the medium) immediately after sterilization (121 °C, 20 min). Sterile polystyrene Petri dishes with vents (JSC Perint, Saint Petersburg, Russia) containing BFM agar were plated and placed in BD GasPak™ anaerobic containers (BD Biosciences, Franklin Lakes, NJ, USA). The incubation was carried out at 37 °C. The measurements were performed in triplicate. The specific growth rate was calculated as the slope of the log_10_ bacterial count.

### 2.4. Quantification of Organic Acid

High-performance liquid chromatography (HPLC) was applied for organic (lactic and acetic) acid detection and quantification as described previously [35], with some modifications. The samples were analyzed on an Agilent 1220 Infinity chromatographic system (Agilent, Santa Clara, CA, USA) coupled with a refractometric detector. The column was an Agilent Hi-Plex H (250 × 4.6 mm). The supernatant of the samples (12,000 rpm for 15 min) was additionally filtered using a cellulose acetate membrane (HAWP, MF-Millipore, St. Louis, MO, USA) with a 0.45 μm pore size. The analysis was performed at 50 °C and a flow rate of 0.3 mL/min 0.002 M H_2_SO_4_ (mobile phase) with an injection volume of 3 µL. The organic acids were diluted in 0.002 M H_2_SO_4_ to concentrations of 1, 5, and 10 mg/mL to prepare the organic acid calibration solution. The qualitative identification was carried out according to the retention time, and the external standard method and the chromatographic peak squares were applied for quantification.

### 2.5. Carbohydrate Assay

The OF monomers (glucose and fructose) and homologs with different degrees of polymerization were detected using capillary electrophoresis (HPCE) as described previously [36,37], with some modifications. The assay was carried out using the Capel−105M system (Lumex^®^, Saint Petersburg, Russia) with a quartz capillary (75 cm length and 50 μm internal diameter). The supernatants of the samples after centrifugation (8500 rpm, 15 min) were additionally purified to remove protein impurities on a centrifugal filter unit (Amicon^®^ Ultra-4 3 kDa, Merck Millipore Ltd., Carrigtwohill, Ireland) by centrifugation at 5500 rpm at 10 °C for 20 min. A solution of 0.5 mM tetradecyltrimethylammonium bromide and 25 mM pyridine-2,6-dicarboxylic acid (both from Lumex^®^, Saint Petersburg, Russia) in 170 mM NaOH was used as the background electrolyte. The temperature was 20 °C and the wavelength was 230 nm for indirect photometric detection of carbohydrates.

### 2.6. Models and Calculations

To describe the growth kinetics of *Bifidobacterium* during continuous cultivation, several factors were taken into account. Firstly, at low flow rate, some of the cells will die due to suppressing conditions. Therefore, at each moment, there will be a dynamic balance between the total number of cells ($X_{Bif}$, cells/mL) and the number of living (L, cells/mL) and dead (*M*, cells/mL) cells according to the following expression:

(1) $$X_{Bif}=L+M.$$

Secondly, the population dynamics of each subpopulation will be determined by the following rate constants [38]: specific growth rate ($\mu_{Bif}$, h^−1^) and death rate ($k_{d\;Bif}$, h^−1^). Moreover, both living and dead cells will be washed out according to the dilution rate (D). This state can be described by the following system of equations:

(2) $$\{\begin{array}{c} \begin{array}{c} \frac{dX_{Bif}}{dt}=\mu_{Bif}\cdot L-D\cdot X_{Bif}\\ \frac{dL}{dt}=\mu_{Bif}\cdot L-D\cdot L-k_{d\;Bif}\cdot L\\ \frac{dM}{dt}=k_{d\;Bif}\cdot L-D\cdot M \end{array} \end{array}.$$

Thirdly, the growth of *Bifidobacterium* will be limited by the carbon substrate (*S*), which can be described by the Monod equation [39]. The growth inhibition by metabolic products (lactic and acetic acids) should also be taken into account [28]. Then, the final equation for the specific growth rate can be written as follows:

(3) $$\mu_{Bif}=\mu_{Bif\;max}\cdot \frac{S}{S+K_{S\;Bif}}\cdot \frac{K_{i\;Bif\;L}}{K_{i\;Bif\;L}+LA}\cdot \frac{K_{i\;Bif\;A}}{K_{i\;Bif\;A}+AA},$$

where $\mu_{Bif\;max}$ is the maximum specific growth rate of *Bifidobacterium* (h^−1^);  S, LA, and AA are the concentrations of substrate, lactic acid, and acetic acid in the bioreactor, respectively (mg/mL), $K_{S\;Bif}$ is the saturation constant (Monod) (mg/mL), and $K_{i\;Bif\;L}$ and $K_{i\;Bif\;A}$ are the growth inhibition constants of *Bifidobacterium* by lactic and acetic acids, respectively (mg/mL).

Fourthly, it was assumed that the rate of death will also be determined by the concentrations of metabolic products. Since it was difficult to find the corresponding equation in the literature, the following expression is proposed:

(4) $$k_{d\;Bif}=k_{d\;Bif_{max}}\cdot \frac{LA}{K_{aLA}+LA}\cdot \frac{AA}{\begin{array}{c} K_{aAA}+AA \end{array}},$$

where $k_{d\;Bif_{max}}$ is the maximum rate of *Bifidobacterium* death (h^−1^), and $K_{aLA}$ and $K_{aAA}$ are the proposed activation constants for the death of *Bifidobacterium* by lactic and acetic acids, respectively (mg/mL), with a similar biological meaning to the saturation constant in the Monod equation.

Lastly, the concentrations of the substrate and products in the fermenter will not immediately reach a steady state, and their change can be described by standard equations [40].

(5) {−dSdt=μBif·YSX·L−D(Sin−S) dLAdt=YLS·dSdt−D·LAdAAdt=YAS·dSdt−D·AA

Here, YSX is the yield of substrate/biomass (economic coefficient of substrate consumption by *Bifidobacterium*) (mg/CFU), $S_{in}$ is the substrate concentration in the medium at the fermenter inlet (mg/mL), and YLS and YAS are the economic coefficients for the formation of lactic and acetic acids from the consumed substrate (mg/mg).

Thus, the growth of *Bifidobacterium* under conditions simulating the intestine (at a low dilution rate) can be described by the following system of equations:

(6) {dXBifdt=μBif·L−D·XBifdLdt=μBif·L−D·L−kd Bif·LdMdt=kd Bif·L−D·M−dSdt=μ·YSX·L−D(Sin−S) dLAdt=YLS·dSdt−D·LAdAAdt=YAS·dSdt−D·AAμBif=μBif max·SS+KS Bif·Ki Bif LKi Bif L+LA·Ki Bif AKi Bif A+AAkd Bif=kd Bifmax·LAKaLA+LA·AAKaAA+AA.

The dilution rate is a constant, and the economic coefficients for the formation of lactic and acetic acids can be found directly from experimental data. However, the analytical solution of Equation (6) is difficult; therefore, to search for a numerical solution, the system was transformed into the integral form of sequentially calculated equations.

(7) μBift+1=μBif max·StSt+Ks·Ki Bif LKi Bif L+LAt·Ki Bif AKi Bif A+AAtkd Bift+1=kd Bifmax·LAtKaLA+LAt·AAtKaAA+AAtXBift+1=Lt·exp(μBif t+1·Δt)−D·XBiftLt+1=Lt·exp(μBif t+1·Δt)−D·Lt−kd Bift+1·LtMt+1=kd Bift+1·Lt−D·MtSt+1=St+[D·(Sin−St)−μBift+1·YSX·Lt+1]·ΔtLAt+1=YLS·(Sin−St+1)−D·LAt·Δt+LA0AAt+1=YAS·(Sin−St+1)−D·AAt·Δt+AA0

Here, Δt is the time step (accepted as 0.1 h), and the indices of the variables correspond to the moment of time at the given iteration step t or next step t+1, which differs from Δt, or at the initial moment of time (0).

The bee colony method was used to determine the equation constants [41,42].

The root-mean-square error (*RMSE*) was selected as the criterion for the determination of the optimal values of constants.

(8) $$RMSE=\sqrt{\frac{\sum_{i=1}^{n}{\left(X_{i\;obs}-X_{i\;pred}\right)}^{2}}{n}}$$

Here, $X_{i\;obs}$ and $X_{i\;pred}$ are the observed and predicted values of *Bifidobacterium* count (L), and n is the number of data points for the experiment.

Taking into account the nature of the growth curves of *Bacillus*, the following kinetic model is proposed that describes only the dynamics of the living cell number ($X_{Bc}$):

(9) $$\frac{dX_{Bc}}{dt}=\mu_{Bc}X_{Bc}-DX_{Bc},$$

where $\mu_{Bc}$ is the specific growth rate (h^−1^).

As in a previous static model [30], it was assumed that the inhibitory effect on *Bacillus* is based on the formation of metabolites by *Bifidobacterium*, which reduces the specific growth rate. In contrast to static fermentation, in continuous cultivation at a low flow rate, substrate limitation cannot be ruled out, as in the case of *Bifidobacterium*. Previously, the absence of β-fructofuranosidase in the strain *Bacillus cereus* ATCC 9634 was shown [30], excluding the possibility of competition for a carbohydrate substrate when cocultured with *Bifidobacterium*. It is known that representatives of the species *Bacillus cereus* are capable of degradation of a number of amino acids (histidine, asparagine, glutamine, proline, etc.) [43], which can be assumed as energy substrates. Additionally, taking into account the obtained data, to calculate the specific growth rate, a factor was introduced taking into account the duration of the growth retardation phase (*α*(*t*)). Thus, to calculate the specific growth rate of *Bacillus*, the following formula is proposed:

(10) $$\mu_{Bc}=\mu_{Bc\;max}\cdot \alpha \left(t\right)\cdot \frac{K_{i\;Bc\;L}}{K_{i\;Bc\;L}+LA}\cdot \frac{K_{i\;Bc\;A}}{K_{i\;Bc\;A}+AA}\cdot \frac{AmA}{K_{S\;Bc\;Am}+AmA},$$

where $\mu_{Bc\;max}$ is the maximum growth rate of *Bacillus*, *α*(*t*) is the coefficient for taking into account the lag phase, $K_{i\;Bc\;L}$ is the lactic acid inhibition constant of *Bacillus* (mg/mL), $K_{S\;Bc\;Am}$ is the acetic acid inhibitory constant (mg/mL), $K_{S\;Bc\;Am}$ is the Monod constant for *Bacillus* growth-limiting amino acids (mg/mL), and AmA is the concentration of *Bacillus* growth-limiting amino acids (mg/mL).

To calculate the lag phase coefficient, it is proposed to use a sigmoidal empirical function

(11) $$\alpha \left(t\right)=\frac{A\cdot e^{B\cdot \left(t-t_{\lambda }\right)}}{1+A\left[e^{B\cdot \left(t-t_{\lambda }\right)}-1\right]},$$

where *A* and *B* are empirical constants that determine the steepness of the sigmoid, and $t_{\lambda }$ is the duration of the lag phase.

Taking into account the characteristics of *Bacillus* growth in a mixed culture and the patterns presented above, it can be assumed that the duration of the lag phase depends on the concentration of lactic and acetic acids at the time of contamination (see in the results). However, it is difficult to determine the explicit nature of this dependence, and the literature data on this issue are extremely scarce. Therefore, the below polynomial quadratic equation is proposed. The calculation of the coefficients of this equation based on experimental data using the least squares method allowed obtaining the following equation:

(12) $$t_{\lambda }=C_{L}\cdot LA+C_{A}\cdot AA+C_{LL}\cdot LA^{2}+C_{AA}\cdot AA^{2}+C_{AL}\cdot LA\cdot AA,$$

where $C_{k}$ denotes the coefficients of the regression equation, the calculation of which used the least squares method and experimental data.

The solution of the equations and determination of the constants were carried out using an iteration method with Δ*t* = 0.1 h, as highlighted before.

The mean relative errors for *Bifidobacterium* count, *LA* concentration, and *AA* concentration in the monoculture model and *Bacillus* count in the coculture model were calculated to demonstrate the accuracy of the predictions as follows:

(13) $$\bar{RE}=\frac{\sum_{i=1}^{n}\frac{\left|Z_{i\;obs}-Z_{i\;pred}\right|}{Z_{i\;obs}}}{n},$$

where $Z_{i\;obs}$ and $Z_{i\;pred}$ are the observed and predicted values of the variable in question (*Bifidobacterium* count (L), *LA* concentration, and *AA* concentration in monoculture model and *Bacillus* count in coculture model), and n is the number of data points for the experiment.

### 2.7. Statistical Analysis

Each experimental point is presented as the mean ± SD of three repetitions. Changes in data were assessed among different experiments using one-way repeated measure analysis of variance (ANOVA). Additionally, to assess the significant differences (*p* < 0.05) among the steady states (stationary phases) two-way ANOVA (for time and experiment conditions factors) with post hoc Tukey honestly significant difference (HSD) test was performed. The samples (subsets) of means log(CFU/mL) for stationary phase points of *Bacillus* monoculture and *Bacillus* coculture with *Bifidobacterium* as well as for the *Bifidobacterium* monoculture were compared. The MatLab software was applied.

## 3. Results

### 3.1. Bifidobacterium Monoculture Experiments and Model

**Figure 2:**
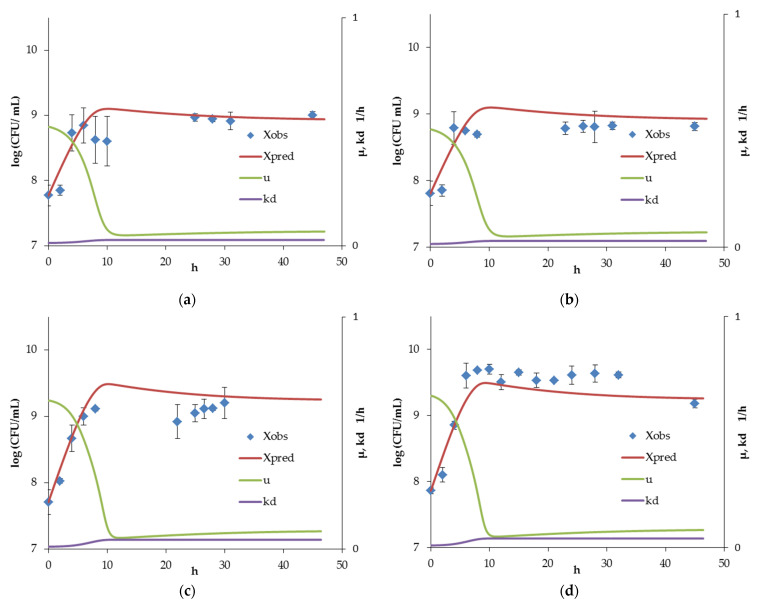

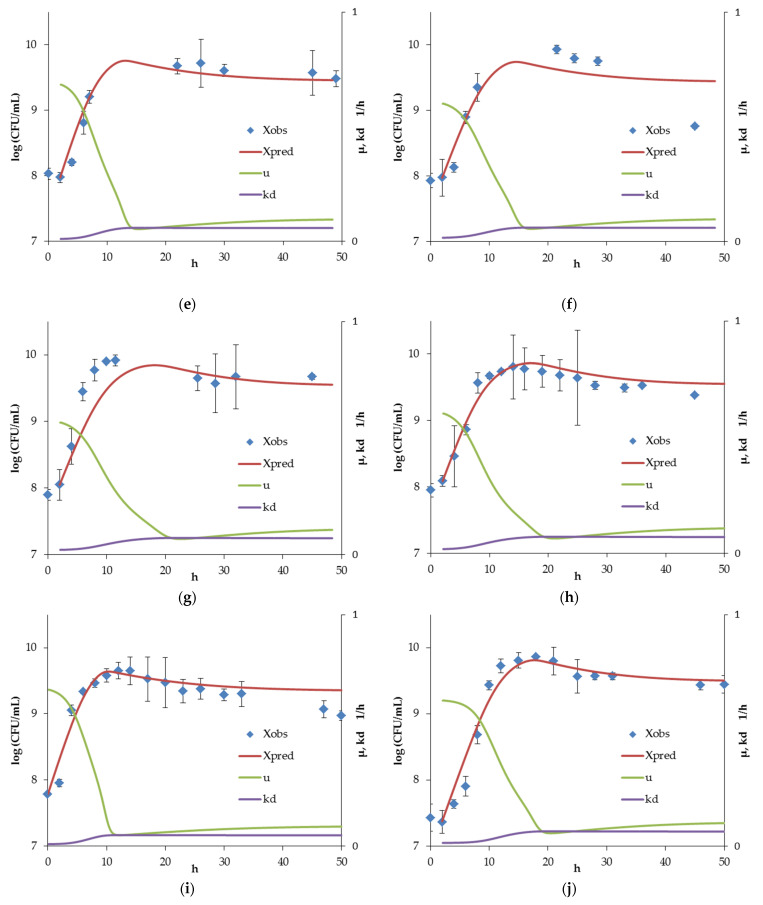
Experimentally observed (*X_obs_*) and model-predicted (*X_pred_*) count of *Bifidobacterium*, along with calculated specific growth rates (µ) and death rate constants (*k_d_*) in monoculture under conditions simulating the distal intestine, at D = 0.04 h^−1^ and OF concentrations of 2 (**a**,**b**), 5 (**c**,**d**), 10 (**e**,**f**), 15 (**g**,**h**), 7 (**i**), and 12 mg/mL (**j**).

The inoculation of *Bifidobacterium* into a sterile nutrient medium was conditionally assumed as a probiotic therapy. If we take into account the high β-fructofuranosidase activity of the strain, then the assumption regarding the predominant consumption of OF can be considered quite reasonable. At an OF concentration of 2 g/L, the number of viable *Bifidobacterium* cells was significantly (*p* < 0.01) lower than that at a concentration of 7 g/L or more (Figure 2). At the same time, no noticeable difference between stationary counts of *Bifidobacterium* was observed at OF concentrations of ≥7 g/L. Thus, the given OF concentration, as can be assumed, was not a limiting substrate for biomass accumulation. In all cases, it can be noted that the population of *Bifidobacterium* reached its maximum count, after which a gradual decline and steady-state occurred.

**Figure 3:**
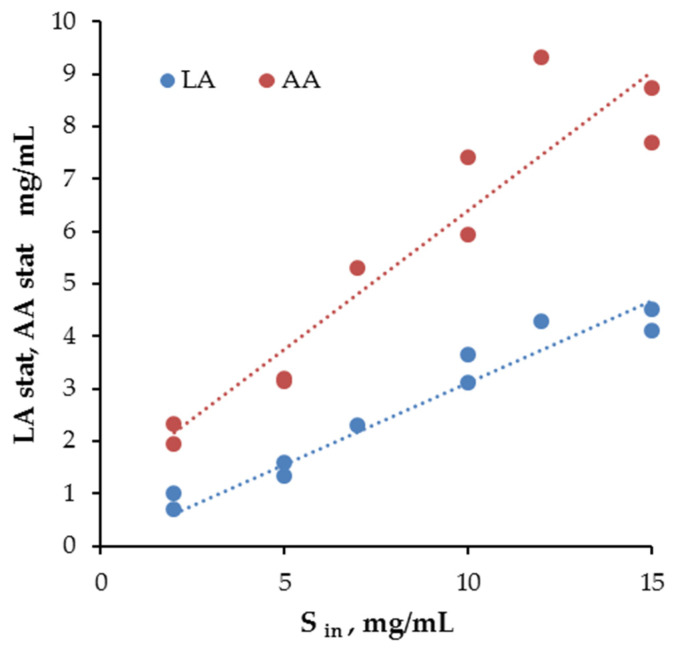
The correlations between OF consumption and *LA* and *AA* production at the stationary phase of *Bifidobacterium* monoculture. It was considered that the carbohydrates were consumed completely, and that the consumed substrate was equal to the introduced substrate.

In all experiments, the complete consumption of the carbohydrate substrate was observed by the stationary stage. The residual concentrations of OF homologs (glucose, fructose, sucrose, and the sum of other oligosaccharides) for feed concentrations of 7 and 12 g/L are given in Supplementary Tables S1 and S2 and Figure S1. Furthermore, the dependences between the stationary concentrations of lactic and acetic acids and the amount of consumed OF (Figure 3) were linear (*R*^2^ = 0.94 and 0.84, respectively). This made it possible to calculate the values of the yields of metabolite production from the consumed substrate. Moreover, the sum of the yields was close to 1. Thus, the OF was completely spent on the energy needs of *Bifidobacterium*.

The calculated model parameters are presented in Table 1. The model was able to predict the equilibrium count of *Bifidobacterium* in the microbial community with acceptable accuracy (see Table 2), since this value was chosen as a criterion to determine the optimal values of the coefficients of Equation (6). On the other hand, the estimation of the stationary concentration of acids was not so accurate. The experimentally observed and predicted by the model values of the concentrations of lactic and acetic acids are presented in Supplementary Figure S2.

### 3.2. Bacillus Monoculture and Coculture Experiments and Inhibition Model

The contamination of the system with *Bacillus* was considered a model of intestinal infection of the host organism, previously strengthened by the intake of probiotics. The introduction of the inoculum with cells in the spore phase corresponded to the predominant state of *Bacillus cereus* in food products, and the conditions in the upper intestines were considered unfavorable for spore germination due to low pH and the high concentration of proteolytic enzymes. In the control experiment, the sterile medium was contaminated with spores of *Bacillus*. The initial *Bacillus* counts were varied through the experiments to improve the predictive value of the developed model.

**Figure 4:**
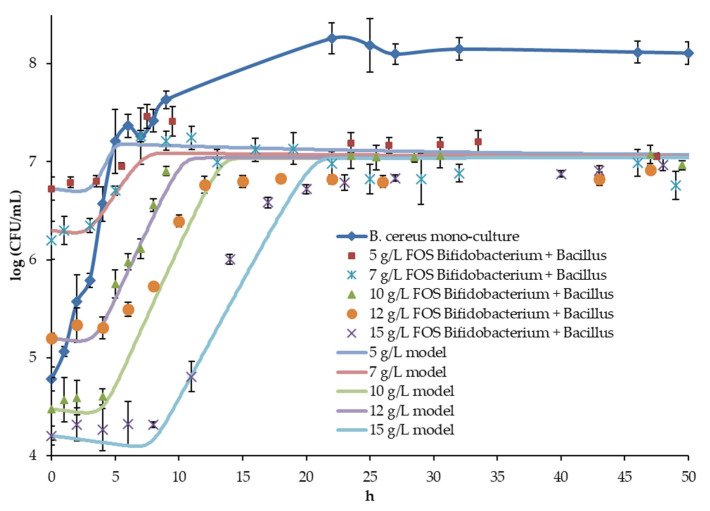
The *Bacillus cereus* cell counts in monoculture and coculture, as well as the coculture model curves.

A significant (*p* < 0.05) difference was noted in the stationary count of *Bacillus* in the control experiment (monoculture) and in the experiments with the inhibitory effect of *Bacillus* (Figure 4). At the same time, the differences were insignificant (*p* < 0.05) for all OF concentrations in culture experiments. Thus, the influence of *Bifidobacterium* on the stationary value of *Bacillus* count is obvious.

The principal diversity was in the noticeable difference in the time interval from infection (*Bacillus* inoculation) to reaching a steady state of *Bacillus* cell count due to differences in the growth lag duration and specific growth rate in the log phase (Table 3).

During model development, the coefficients of the square polynomial regression equation (Equation (12)) were first determined to calculate the growth delay time.

(14) $$t_{\lambda }=-3.46\cdot LA+2.77\cdot AA+2.20\cdot LA^{2}-0.23\cdot AA^{2}-0.56\cdot LA\cdot AA.$$

The F-test showed good correlation (*p* < 0.05). Other parameters of the model (Equations (9)–(11)) were determined numerically, enabling predictions with low error for all coculture experiments (Table 1). The model predicted *Bacillus* growth with acceptable accuracy in all experiments (see Table 2).

## 4. Discussion

In this study, we considered continuous cultivation at low flow rates, where the patterns of culture growth differ from the classical chemostat, in which the consumption of nutrients by cells is compensated for by their intake, and the accumulation of metabolites is compensated for by their removal at equal rates. Although continuous cultivation at low dilution rates has been used in microbiological research for a long time [38], data on the use of the appropriate mathematical expression to describe the intestinal microbial community under similar metabolic conditions are extremely scarce. Studies that used fermentation data from the entire microbial community of the gastrointestinal tract, such as rumen, to obtain numerical values for quantitative model parameters are not numerous [44]. The approach of replacing the entire community with key members (synthetic community) seems to be justified, and it has been applied more widely [25,27,28].

Taking into account the suppressing conditions that occur at low flow rates, the events in a monoculture of *Bifidobacterium* can be considered as the establishment of the dynamic balance between cell growth and cell death, with a gradual decrease in the specific growth rate and increase in the death rate constant. These parameters were included in previously described models [28]; however, it was proposed herein to consider them not as independent variables, but as functions of suppressing factors (substrate and metabolite concentrations). The k_d_ value obtained by Pinto et al. was 0.0139 for *Bifidobacterium* in the synthetic intestinal community, corresponding to an *LA* concentration of ~0.8 mg/mL and *AA* concentration of ~1.5 mg/mL. Furthermore, the growth curve has an extremum before achieving dynamic equilibrium, which can be reflected by the formula:

(15) $$\mu_{Bif}-k_{d\;Bif}-D=0.$$

On the other hand, the oscillation predicted by a number of models [26] has not been experimentally confirmed for either mono or coculture under conditions simulating the distal intestine.

It was assumed that the model would make it possible to accurately predict the stationary concentrations of lactic and acetic acids, since they have a decisive influence on the outcome of microbial infection. However, the prediction accuracy for these functions was lower than for the count of *Bifidobacterium*. In the future, it will be possible to expand the growth model of a *Bifidobacterium* monoculture and determine the optimal values of parameters using acid concentrations as criteria.

Two features are key for the patterns of *Bacillus* growth. Firstly, it is necessary to note the increase in time of the lag phase, which, most likely, is also associated with the spore germination time. Thus, at the maximum prebiotic concentration, this delay was 8 h. Secondly, the specific growth rate of *Bacillus* in the stage of active growth increased when the OF concentration was raised to 10 g/L, while, at high concentrations, the changes were small. This feature requires further research, although it can be assumed that it is associated with the transformation of acetate into Ac-CoA, which has been described, for example, for *Bacillus subtilis* under anaerobic conditions [45].

A correction factor for modeling the lag phase was previously introduced by Baranyi and Roberts [46]. They also proposed to calculate it as a function of the limitation substrate, which was not consistent with the objectives of our study. Therefore, in this work, we used a sigmoid function with an inflection point determined by the duration of the lag phase as a function of the concentration of lactic and acetic acids. Thus, the model should help to predict two key events that can affect the bacterial intestinal infection progress after pathogens enter (in the current study, imitated by inoculation of the system with *Bacillus*), since their combination determines the duration before the pathogen reaches the maximal count. It can be assumed that a longer time interval increases the chances for the microbiota and the host organism to prevent the disease.

The effect of probiotics (*Bifidobacterium longum* and *Lactobacillus fermentum*) and prebiotics (isomaltooligosaccharides and short-chain fructooligosaccharides) on the elderly fecal microbiota [47], as well as on pathogen infection of the microbial community [13], was previously studied. However, in these and other studies, where three-stage continuous fermentation was used, the frequency of sampling was at least several hours, which provides information about the state of the microbial community, but makes it difficult to search for the parameters of a complex mathematical model. Our results are limited, on the one hand, since the studies were carried out with a synthetic community and monocultures. On the other hand, they allowed us to consider events similar to the introduction of probiotics and pathogens into fecal culture with a high resolution. A final statement about the predictive value of the models can be made after the transfer of the experiment to fecal three-stage continuous fermentation.

## 5. Conclusions

The study determined the main patterns of development of *Bifidobacterium* and the outcome of bacterial infection under conditions simulating the distal intestine at various doses of the prebiotic substance administered. The concentration of carbohydrates strictly determined the production of metabolites, but the limitation of *Bifidobacterium* growth was observed only at extremely low values. Significant differences in the growth delay time and specific growth rate of *Bacillus* were established depending on the concentration of lactic and acetic acids at the contamination time. Mathematical models based on kinetic laws were developed to predict the behavior of individual members of the intestinal microbial community. Although the considered model cannot give definitive answers regarding the outcome of intestinal infection in vivo, a number of the obtained patterns are important for understanding key events. In the future, it seems promising to test the model in a three-stage continuous system with fecal culture.

## Figures and Tables

**Table 1:** The model parameter values.

Parameter Description	Unit	Value
*Bifidobacterium* monoculture		
$\mu_{Bif\;max}$	h^−1^	1.003
$K_{S\;Bif}$	mg/mL	0.727
$K_{i\;Bif\;L}$	mg/mL	5.46
$K_{i\;Bif\;A}$	mg/mL	5.33
$k_{d\;Bif_{max}}$	h^−1^	0.117
$K_{aLA}$	mg/mL	2.12
$K_{aAA}$	mg/mL	2.05
YSX	mg/cell	1.40 × 10^−6^
YLS	mg/mg	0.313
YAS	mg/mg	0.631
*Bacillus* coculture		
$\mu_{Bc\;max}$	h^−1^	1.214
$K_{i\;Bc\;L}$	mg/mL	8.85
$K_{i\;Bc\;A}$	mg/mL	10.15
$K_{S\;Bc\;Am}$	mg/mL	0.18
AmA	mg/mL	0.71
A	-	0.55
B	-	2.23

**Table 2:** The mean relative errors values (%) for *Bifidobacterium* monoculture model and *Bacillus* coculture model with *Bifidobacterium*. The relative errors compare observed (experimental) and predicted data.

Inlet OF Concentration	*Bifidobacterium* Monoculture			*Bacillus* Cell Count in Coculture
(mg/mL)	Viable Cell Count	*LA*	*AA*	
2	1.93	27.2	19.3	-
2	2.17	68.9	10.3	-
5	2.03	24.4	24.8	-
5	2.11	33.1	25.3	0.75
7	2.10	27.5	10.5	0.93
10	1.14	9.8	18.3	-
10	2.58	7.39	5.66	2.78
12	2.12	13.7	11.9	0.75
15	2.39	29.9	18.9	-
15	1.00	14.2	27.5	0.74

**Table 3:** Specific growth rates of *Bacillus cereus* in the log phase and duration of the log phase during cocultivation with *Bifidobacterium adolescentis* while varying OF concentration.

*S_in_* (mg/mL)	*LA* (mg/mL)	*AA* (mg/mL)	Total Acids (mg/mL)	$\mu_{Bc}$ at log Phase (h^−1^)	$t_{\lambda }\;(h)$
Monoculture				0.78	0
5	1.23	3.44	4.67	0.38	3.5
7	1.03	6.60	7.63	0.53	3.0
10	3.20	8.02	11.22	0.66	3.5
12	3.22	8.28	11.50	0.59	4.0
15	3.54	7.49	11.03	0.62	8.0

## Data Availability

Data are contained within the article and supplementary materials.

## Supplementary Materials

The following supporting information can be downloaded at https://www.mdpi.com/article/10.3390/microorganisms10050929/s1: Figure S1. Electropherogram for the determination of oligosaccharides during cultivation of *Bifidobacterium adolescentis* at OF inlet concentration of 12 mg/mL at 0 h of growth; Figure S2. Experimentally observed (*LA_obs_* and *AA_obs_*) and model-predicted (*LA_pred_* and *AA_pred_*) concentrations of lactic acid (*LA*) and acetic acid (*AA*) in the *Bifidobacterium* monoculture under conditions simulating the distal intestine, at D = 0.04 h^−1^ and OF concentrations of 2 (a, b), 5 (c, d), 10 (e, f), 15 (g, h), 7 (i), and 12 mg/mL (j); Table S1. The concentration of carbohydrates during continuous cultivation of *Bifidobacterium adolescentis* at OF concentration of 7 mg/mL; Table S2: The concentration of carbohydrates during continuous cultivation of *Bifidobacterium adolescentis* at OF concentration of 12 mg/mL.
