# Supplementary material for: A Study and Modeling of Bifidobacterium and Bacillus Coculture Continuous Fermentation under Distal Intestine Simulated Conditions
Source: Microorganisms. 2022 Apr 28;10(5):929. doi: 10.3390/microorganisms10050929 (PMC9147766; doi:10.3390/microorganisms10050929)
Supplement: Supplementary file 1 [file microorganisms-10-00929-s001.zip › microorganisms-1639504-supplementary.pdf]

Table S1. The concentration of carbohydrates during continuous cultivation of *Bifidobacterium adolescentis* at OF concentration of 7 mg mL^{-1}.

Time h	Fructose μg mL^{-1}	Glucose μg mL^{-1}	Sucrose μg mL^{-1}	Sum of fructans μg mL^{-1}	Number of homologs
0	1302	71.42	373.9	1747.32	8
2	1275	-	121.1	1396.1	6
14	55.12	-	-	55.12	3
23	-	-	46.29	46.29	1
54	34.96	-	-	34.96	3
75	8.975	-	-	8.975	4

Table S2. The concentration of carbohydrates during continuous cultivation of *Bifidobacterium adolescentis* at OF concentration of 12 mg mL^{-1}.

Time h	Fructose μg mL^{-1}	Glucose μg mL^{-1}	Sucrose μg mL^{-1}	Sum of fructans μg mL^{-1}	Number of homologs
0	1944	101	498	2543	9
6	2058	63.67	476.4	2598.0	9
10	2230	-	455.3	2685.37	9
25	33.44	-	-	33.44	3
50	37.66	-	-	37.66	3
98	39.24	-	-	39.24	3

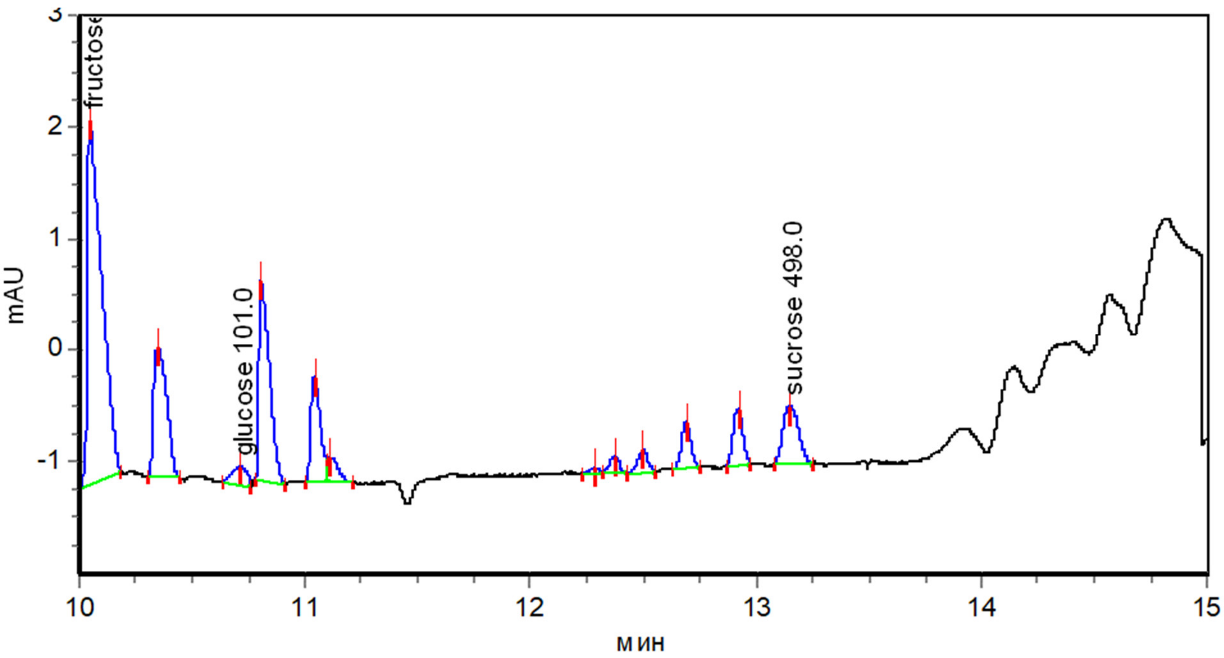

Figure S1. Electropherogram for the determination of oligosaccharides during cultivation of *Bifidobacterium adolescentis* at OF inlet concentration of 12 mg mL^{-1} at 0 hours of growth.

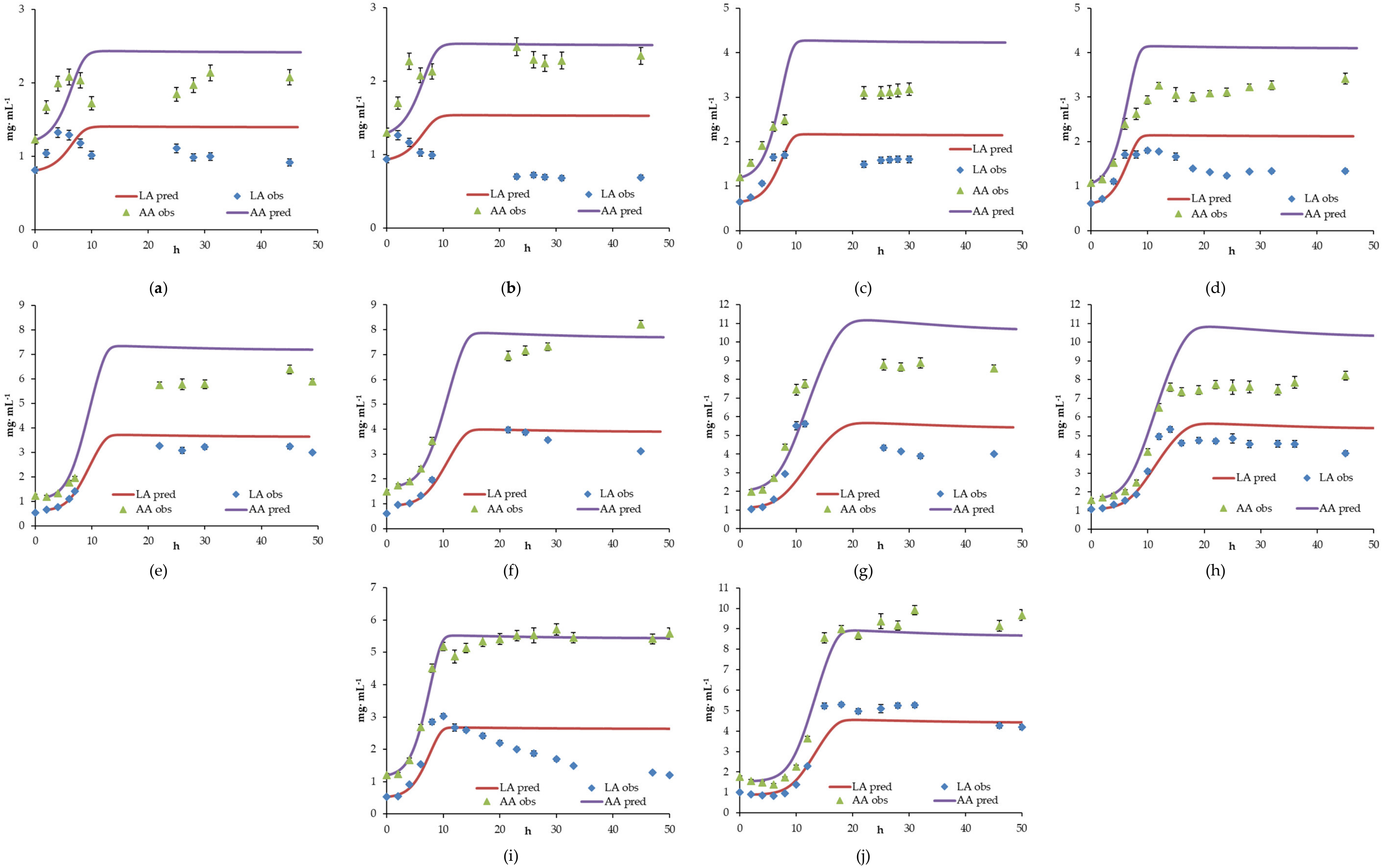

Figure S2. Experimentally observed (LA_{obs} and AA_{obs}) and predicted by the model (LA_{pred} and AA_{pred}) concentrations of lactic acid (LA) and acetic acid (AA) in the bifidobacteria monoculture under conditions simulating the distal intestine, at D = 0.04 h^{-1} and concentrations OF 2 mg mL^{-1} (a, b); 5 mg mL^{-1} (c, d); 10 mg mL^{-1} (e, f); 15 mg mL^{-1} (g, h); 7 mg mL^{-1} (i); and 12 mg mL^{-1} (j).
